# Adverse psychosocial working conditions and minor psychiatric disorders among bank workers

**DOI:** 10.1186/1471-2458-10-686

**Published:** 2010-11-10

**Authors:** Luiz S Silva, Sandhi M Barreto

**Affiliations:** 1 Faculty of Medicine, Federal University of Minas Gerais, Belo Horizonte, Brazil; 2 Banco do Brasil SA, Belo Horizonte, Brazil

## Abstract

**Background:**

In most countries, the financial service sector has undergone great organizational changes in the past decades, with potential negative impact on bank workers' mental health. The aim of this paper is to estimate the prevalence of minor psychiatric disorders (MPD) among Brazilian bank workers and to investigate whether they are associated with an adverse psychosocial working environment.

**Methods:**

A cross-sectional study of a random sample of 2,500 workers in a Brazilian state bank in 2008. The presence of MPD was determined by the General Health Questionnaire.(GHQ). Psychosocial work conditions were assessed by means of the Effort-Reward Imbalance (ERI) and Job Content Questionnaire (JCQ). The presence and magnitude of the independent associations between MPD and adverse psychosocial working conditions were determined by Prevalence Ratios, obtained by Poisson regression.

**Results:**

From 2,337 eligible workers, 88% participated. The prevalence of MPD was greater among women (45% vs. 41%; p > 0.05). In the multivariate analysis, the prevalence of MPD was twice as high among bank workers exposed to high psychological demand and low control at work and under high effort and low reward working conditions. The lack of social support at work and the presence of over-commitment were also associated with higher prevalence of MPD. A negative interaction effect was found between over-commitment and effort-reward imbalance.

**Conclusion:**

The prevalence of MPD is high among bank workers. The results reinforce the association between MPD and adverse psychosocial working conditions, assessed by the JCQ and ERI models. The direction of the interaction observed between over-commitment and ERI was contrary to what was expected.

## Background

Globalization and market deregulation have resulted in substantial restructuring in the financial services sector and in the way work is organized and done over the last few decades, in both industrialized and developing countries. A report by the International Labor Organization shows that this modernization has led to the development of a number of concerns for financial service workers, such as increasing time pressure, excessive work demands, role conflict, ergonomic insufficiencies, problematic customer relations and an increase in reported cases of stress and violence [1].

Work-related stress can affect individuals when their coping mechanisms or abilities to control the demands placed on them become ineffective or worn out [2]. It has been linked to a range of adverse physical and mental health outcomes, including depression and anxiety, as well as maladaptative behaviors such as drinking and smoking [3]. Rapid changes in work practices and job insecurity were prospectively associated with mental health problems among civil servants in London [4]. In Brazil, there was a sizeable increase in the number of leaves of absence due to minor psychiatric disorders among workers following the implementation of restructuring changes in the bank sector [5].

Given the conceptual difficulties involving the study of mental health, there is a growing use of indicators such as minor psychiatric or non-psychotic disorders. These disorders indicate a certain affect on the worker's psychological life structure, hence in the interrelations with his/her social and working lives [6]. Minor psychiatric disorders have been prospectively associated with increased sickness absence at work [7], incidence of coronary heart disease [8] and long term suicide risk [9].

The effects of job stress on a variety of mental health outcomes have been widely studied using the demand-control [10] and effort-reward imbalance [11] theoretical models [12]. A review of empirical studies shows that the stressful aspects of work measured by these two models are different, and the adverse health effects are independent of each other, which suggests that the two models are complementary [10]. While the job demand-control model emphasizes task-level control, the effort-reward imbalance model emphasizes the rewards given to employees

Robert Karasek's model [13] separates four types of working experiences generated by the interaction of the psychological demand and control levels: low-strain (low demand and high control), active (high demand and high control), passive (low demand low control), and high-strain (high demand and low control). As their central assumption, negative reactions to psychological demands (exhaustion, stress, depression, and other physical ailments) occur in jobs where there are high demand and low control. The presence of social support may be evaluated by adding this third and important dimension to the model. It is assumed that health and well-being decrease in the so-called *iso-strain *situation, where high demand, low control and low social support at work co-exist [14-16]. Though widely accepted, results from studies that investigated this hypothesis are still inconsistent [14].

Johannes Siegrist's model [17] postulates that jobs characterized by an imbalance characterized by great effort and low reward are extremely stressful and can bring about health problems. The model assumes, still, that a person with a motivational pattern to work excessively and with great need of reward will respond inflexibly to the imbalance between effort and rewards at work, being the most stressed and prone to getting ill. This over-commitment pattern is analyzed as the intrinsic hypothesis of the model. Although there is still no sufficiently established empirical evidence, the hypothesis is that over-commitment has a moderating effect, modifying the association between the imbalance between effort and reward and the worker's health [10,11,15].

Similar to changes in banking sector worldwide, Brazilian banks underwent significant organizational change in the past few decades, with major impact on employees' working conditions. In Brazil, such changes are associated with significant increases in the morbidities such as musculoskeletal work-related and mental disorders. In 2003, about 21% of the sick leave and 49% of the invalidity retirements in a major Brazilian bank were due to mental health problems [5], with increasing prevalence of these disorders[18].

The study aims to estimate the prevalence of minor psychiatric disorders in workers of a large bank in Brazil and to investigate if they are associated with adverse psychosocial working conditions according to JCQ and ERI models. It also tests whether these associations are modified by social support at work or the presence of over-commitment, as proposed by the JCQ and ERI models, respectively.

## Methods

All of the 40,005 employees of a large Brazilian state bank who worked in any of the 27 capitals and the Federal District at the end of 2007 were eligible to participate. A simple random sample of 2,500 workers, stratified by sex, was drawn using the bank payment roll. This list was released after the ethical approval of the project and with a signed agreement of the authors.

The adequacy of the questionnaire, as well as the data collection procedures, were tested in a pilot study with 100 eligible individuals from all over the country who did not take part of the sample selected for the study. The authors sent the questionnaire with a letter explaining the research and inviting the workers to participate. The information was obtained using a self-applied structured questionnaire, sent by the authors, with a letter explaining the research and inviting the workers to participate. It was sent by post (97%), and by e-mail (3%). Confidentiality was assured to employees by a detailed letter signed by the investigators explaining the purpose of the study and its ethics commitments. The confidentiality was also explicit assured in the free informed consent form. The data were collected between August 2008 and December 2008.

Presence of MPD was assessed using a twelve-question version of the General Health Questionnaire (GHQ-12) adapted by Mari and Williams [19]. It is a commonly-used screening instrument to identify individuals with minor psychiatric illness in population and work-environment studies. The shortest version of the questionnaire has been extensively validated and used in a number of countries and in different languages. The presence of minor psychiatric disorders was defined by the cut point equal to or higher than 4 in the GHQ final score [19,20].

The psychosocial factors at work were assessed by means of two tools: the reduced version of the JCQ, adapted to Portuguese in 2003 by Araújo [21] and the ERI scale, adapted to Portuguese by Silva & Barreto [22].

JCQ contains 22 questions with answer options in Likert scale (1-4), varying from "strongly agree" to "strongly disagree". The block regarding social support contains eight questions about the relationship with colleagues and managers with four answer options also varying from "strongly agree" to "strongly disagree". Answers were coded according to the *Job Content Questionnaire User's Guide *(Karasek, 1985) [23]. Based on the assumptions gathered in Karasek's model, the variables were dichotomized by the median value, combined in four distinct categories. Workers exposed to a combination of high demand and low control were considered as the highest exposure group. Active job (high demand and high control) and passive job (low control and low demand) were regarded as intermediate groups. 	Those reporting high control and low demand (low demand job) were the reference category in the statistical analysis. The final scores for demand sub-scale range from 6 (minimum value) to 48 (maximum value). For the control subscale, final scores vary from 24 and 96.

ERI assembles three one-dimensional scales: effort (6 items), reward (11 items) and over-commitment (6 items). The reward scale can be subdivided in three subscales: esteem, job security and job promotion/salary. The analysis was based on the comparison of the tertiles of the effort and reward subscales, dichotomizing the variables in the higher tertile and building four independent categories: low effort/high reward (reference), high effort/high reward, low effort/low reward and high effort/low reward (greater exposure). Based on the theoretical assumptions of these two models, working stress indicators were also built for the DC model (demand over control), using the median of the distribution, and for the ERI model (effort over reward) using the highest tertile of the distribution as a cut point.

The questionnaire also included sociodemographic, health and psychosocial information. Sociodemographic variables included: sex, age (20 - 70 years), marital status, having children, schooling, ethnic group, job duration (1-37 years) and occupational category. Behavioral variables were: smoking, use of alcoholic beverages (one or more doses in the past 14 days). Health variables were report of medical diagnosis of chronic and heart diseases (hypertension, diabetes, asthma/bronchitis, myocardial infarction, stroke and musculoskeletal disorders). Psychosocial variables were: exposure to stressful situations in the past year (being robbed, loss of a loved person, financial difficulties, hospitalizations, breaking up of relationships, unwanted change of address) and exposure to prejudice in the past year regarding race, gender, sexual orientation, religion, disability, age or socio-economical condition. All these co-variables were considered potential confounding factors in the association between MPD and adverse job conditions defined by JCQ and ERI models.

The prevalence of mental disorder was assessed for each sex and categories defined according to JCQ and ERI scales. Different weights were attributed for men and women in the pooled analysis in order to correct for the difference in the probability of each sex participating in the study. The *χ*^2 ^test with 5% significance level was used to test the differences between presence of MPD and the variables of interest and potential confounders. Given the high prevalence of the dependent variable, we used prevalence rate ratios (PR) instead of odds ratio to assess the magnitude of the statistical associations between MPD and adverse psychosocial working conditions to avoid overestimation of effect sizes. Prevalence ratios were obtained by Poisson regression, with a robust variance, with a 95% confidence interval [24,25]. The analysis was performed with Stata 9.2 (Stata Corp., College Station, TX, USA).

Two separate multivariate analysis were run, one including the JCQ categories and other including the ERI categories. The two models were not adjusted for each other representing completely distinct categories. All the variables associated with MPD in the univariate analysis at the level of p < 0.20 were considered in the multivariate analysis. Only the variables which remained associated at the level of p < 0.05 were retained in the final analysis. Finally, the interaction between excess demand-control and social support were tested by means of adding an interaction term to the final adjusted JCQ model. The interaction between effort-reward imbalance and over-commitment was tested by adding an interaction term to the final adjusted ERI model.

This study was approved by the Research Ethics Committee of the Federal University of Minas Gerais and all participants signed the free informed consent form.

## Results

Of the total 2,500 participants selected for the study, 163 were ineligible due to retirement, long term sick leave or temporary suspension of the job contract. The characteristics of the 283 workers not participating in the survey did not differ significantly from participants regarding sex (p = 0.758), age (p = 0.282), marital status (p = 0.758), schooling (p = 0.256) and job duration (p = 0.481). Of the 2,337 eligible workers, 88% participated in the study.

Most men and women were in their forties, married, university graduates, employed at the company for 15 to 24 years, self declared white, non-smokers and alcoholic beverage users. The most frequent disease reported by participants was hypertension (28%), followed by bronchitis (23%) and work-related musculoskeletal disorders (23%).

The overall prevalence of MPD was 43%, being 41% among men and 45% among women (p > 0.05).

In the univariate analysis (Table 1), having children, smoking, presence of heart or any chronic disease, exposure to stressful situations or to any kind of prejudice were statistically associated with MPD.

**Table 1:** Prevalence and prevalence ratios of minor psychiatric disorders among bank workers according to sociodemographic, life habits, health factors and exposure to stressful and discriminatory events Brazil, 2008.

Variable	Categories	N	MPD*(%)	PR** (IC 95%)
Gender	Male	1021	41	1.00
	Female	1033	45	1.10(0.99 - 1.21)
Age(years)	20-29	346	40	1.00
	30 - 39	600	40	1,00(0.85 - 1.19)
	40 - 49	739	48	1.24(1.06 - 1.44)
	50 - 59	369	41	1.03(0.86 - 1.24)
Schooling	High School	422	42	1.00
	University Graduate	1228	43	1.01(0.88 - 1.15)
	University Post-Graduate	404	45	1.06(0.90 - 1.24)
Marital Status	Married	1021	42	1.00
	Single	870	43	0.96(0.86 - 1.07)
	Separated/Divorced	163	48	1.10(0.92 - 1.33)
Children	No	959	46	1.00
	Yes	1095	41	1.12(1.01 - 1.24)
Race/Skin Color	White	1488	43	1.00
	Black	491	44	0.89(0.66 - 1.20)
	Other	75	39	1.04(0.92 - 1.17)
Smoker	No	1735	40	1.00
	Yes	319	57	1.45(1.29 - 1.62)
Alchohol intake in	No	601	41	1,00
Past 14 days ***	Yes	1453	44	1.05(0.92 - 1.21)
Exposure to stressful	No	1444	41	1.00
Situations (past year)	Yes	610	48	1.16(1.04 - 1.29)
Exposure to prejudice	No	1761	41	1.00
(past year)	Yes	293	54	1.31(1.16 - 1.48)
Heart disease	No	1480	39	1.00
	Yes	574	54	1.39(1.25 - 1.54)
One or more chronic	No	1391	37	1.00
Diseases	Yes	663	56	1.53(1.39 - 1.69)
Duration of	0 - 5	482	40	1.00
Employment (years)	6 - 14	593	44	1.10(0.95 - 1.28)
	15 - 24	510	45	1.13(0.97 - 1.31)
	25 and more	469	43	1.08(0.93 - 1.27)

*MPD: minor psychiatric disorders, assed by GHQ-12 > 4

** PR: Prevalence Ratio by Poisson regression

*** one or more doses per day

**Figure 1:**
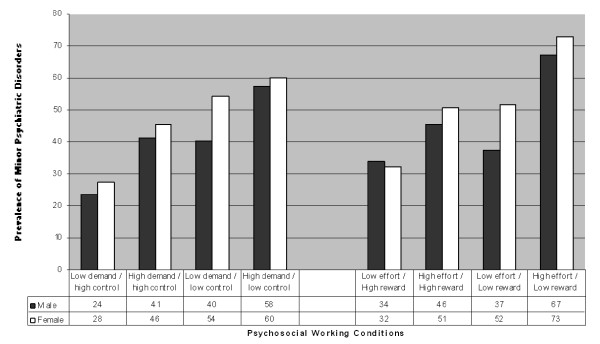
**Prevalence (in percentage) of Minor Psychiatric Disorders among bank workers by gender and according to the categories defined by the Demand-Control and Effort-Reward scales**. Brazil, 2008.

Figure 1 and Table 2 indicates that adverse working conditions assessed by both scales were statistically associated with the presence of MPD. Compared to workers exposed to low-demand and high-control activities, the prevalence of MPD more than doubled among those in maximum demand and minimum control conditions. The same is observed regarding ERI, with the prevalence of MPD shifting from 33% among those in low-effort and high-reward working condition to 70% among workers with high effort and low reward. Both the absence of social support at work and the presence of over-commitment were also statistically associated with the presence of MPD among participants.

**Table 2:** Prevalence and prevalence ratios of minor psychiatric disorders among bank workers according to working characteristics and psychosocial conditions.

Variable	Categories	N	MPD* (%)	PR** (IC 95%)
Occupational	Manager	528	44	1.00
Category	Teller	142	44	1.21(0.97 - 1.50)
	Assistant	652	46	1.22(1.06 - 1.40)
	Analist	183	43	1.13(0.92 - 1.39)
	Clerk	548	38	1.17(1.01 - 1.36)
				
Work conditions	Low demand/high control	692	26	1.00
assessed by	High demand/high control	390	44	1.72(1.44 - 2.05)
Demand/Control	Low demand/low control	300	48	1.83(1.53 - 2.20)
Scale ***	High demand/low control	672	59	2.32(2.01 - 2.69)
				
Social Support at work	Yes	1379	34	1,00
	No	675	62	1.86 (1.68 - 2.05)
				
Work conditions	Low effort/High reward	1032	33	1.00
assessed by Effort-	High effort/High reward	461	48	1.43(1.25 - 1.63)
Reward imbalance	Low effort/low reward	295	46	1.33(1.13 - 1.56)
Scale ****	High effort/low reward	266	70	2.10(1.86 - 2.37)
				
Over-commitment	No	1267	30	1.00
	Yes	787	65	2.25(2.03 - 2.49)

Brazil, 2008.

*MPD: minor psychiatric disorders, assed by GHQ-12 > 4

** PR: Prevalence Ratio by Poisson regression

*** Karasek 1979

***** Siegrist 1996

In the multivariate analysis considering the demand-control model (Table 3), the presence of MPD increased significantly with the worsening of the psychosocial working condition, with negligible reduction in the magnitude of the associations observed in the univariate analysis. The interaction between high demand/low control and the lack of social support at work was not statistically significant (p > 0.05).

**Table 3:** Results of the multivariate analysis of the association between minor psychiatric disorders among bank workers and adverse psychosocial working conditions assessed by the Demand-Control model.

Associated factors	Categories	PR (IC 95%)
Exposure to stressful	No	1.00
situations (past year)	Yes	1.30 (1.17 - 1.46)
		
Exposure to prejudice (past year)	No	1.00
	Yes	1.37 (1.20 - 1.55)
		
Working conditions*	Low demand/high control	1.00
	High demand/high control	1.66 (1.40 - 1.98)
	Low demand/low control	1.59 (1.33 - 1.91)
	High demand/low control	1.85 (1.57 - 2.19)
		
Social support at work	Yes	1.00
	No	1.59(1.41 - 1.80)

Brazil, 2008 (n = 2054)

* Defined by the Demand-Control Model (Karasek, 1979)

The multivariate analysis considering the ERI categories also shows a significant increase in the magnitude of the prevalence ratios as the psychosocial conditions worsen (Table 3). There was a statistically significant interaction between effort-reward imbalance and over-commitment, with the prevalence of MPD reducing in the presence of over-commitment (p < 0.001; Table 4).

**Table 4:** Results of the multivariate analysis on the association between minor psychiatric disorders among bank workers and stressful working conditions assessed by the Effort-Reward Imbalance model.

Associated factors	Categories	PR (IC 95%)
Exposure to stressful situations (past year)	No	1.00
	Yes	1.16(1.05 - 1.29)
		
Exposure to prejudice (past year)	No	1.00
	Yes	1.21(1.06 - 1.38)
		
Working conditions*	Low effort/High reward	1.00
	High effort/High reward	1.16 (1.02 - 1.32)
	Low effort/Low reward	1.33 (1.14 - 1.55)
	High effort/Low reward	1.46 (1.29 - 1.66)
		
Over-commitment	No	1.00
	Yes	2.13 (1.90 - 2.38)
		
Effort-reward imbalance **	vs Over-commitment	0.70(0.56 - 0,86)

Brazil, 2008. (n = 2054)

* Defined by the Effort-Reward Model (Siegrist, 1996).

** Effort/Reward Imbalance defined by the upper tertile of the Effort/Reward Ratio.

## Discussion

The results show that MPD is highly prevalent among bank workers being more common among those exposed to adverse psychosocial working conditions, assessed by means of demand-control and ERI scales. The absence of social support at work and the presence of over-commitment were also associated with increased prevalence of MPD. In addition, we found a negative interaction between over-commitment and adverse working conditions defined by the ERI model.

The work has some limitations that include the study population and design, the healthy worker effect and data collection through self-response. It was conducted in one specific bank and the results may not be generalized to other employees in the financial service sector as some features of working conditions, such as job security, vary among different companies. Moreover, the cross-sectional design of this study does not allow one to infer on the causal nature of the associations found between stress at work and common mental disorder. It is not possible, on this basis, to dismiss the presence of reverse causality. As MPD constitute an important cause of temporary leave and invalidity pensions among bank workers in the country [5], it is possible that individuals with severe mental disorders did not participate in this work, thus underestimating the prevalence of MPD in the study population. But, despite the limitations, it analyzed a large sample of bank workers with a high participation rate (88%). Yet, it is important to point out that the results of prospective studies in various occupational categories reinforce the hypothesis of causality between the presence of adverse psychosocial working factors and the development of MPD [7,26].

Finally, both psychosocial working factors and mental disorders were assessed by self-report. Regarding adverse psychosocial working conditions, studies have shown that self-reported stress at work has a good capability of predicting adverse health events [7,26]. Although personality factors may contribute to this association, they do not completely account for it, as show by Stansfeld (1998) [27] after adjustment for negative affectivity and hostility.

Regarding potential confoundings for the association between adverse psychosocial working conditions and MPD, the statistical analysis considered important factors such as gender, age, marital status, schooling, job position and exposure to stressful life events and prejudices. But adjusting for these factors did not change the results found substantially, confirming the independence of the association between MPD and adverse psychosocial working conditions.

As far as we know, this is the first Brazilian study to investigate the psychosocial working environment among bank workers, and GHQ was used here to assess the presence of mental disorder due to its high sensitivity (85%) and specificity (79%) when compared to Clinical Interview Schedule [19] and for being a validated tool, broadly used in studies in Brazil [19,20]. The prevalence of MPD varies according to the population, the tools used, and the moment when the assessment took place. The prevalence of MPD differs considerably among distinct occupational groups, but it is generally smaller than the one observed in this study [20,21].

The prevalence of MPD found in the present work is very high, greatly exceeding the one observed in population-based studies performed in Brazil. Multi-centered studies estimated that the prevalence of mental disorder in the adult population of Brazil (ages 20 to 69) varies from 19% to 25% [28]. It is also high when compared to international data. A study using the GHQ in Great Britain has also reported a higher prevalence among bank workers than among university professors and public sector workers. However, albeit high, the prevalence found among English bank workers was much lower than that found in this work (28% among women and 25% among men) [4].

In the studied population, the prevalence of MPD did not vary significantly according to gender, marital status, race/skin color, schooling, duration of employment and job position, as described by the review of Doef et al. [29]. For gender, Pearson *χ*^2 ^has the probability of 0.07. This might be explained by the great homogeneity of bank workers regarding these characteristics [7,30-32]. In this company, there is an inclusive policy that equates men and women in terms of job categories and incomes. The distributions of men and women in this work are also similar with regard to educational level, marital status and length of employment.

The results show very clearly that people subjected to adverse psychosocial working environments have higher prevalence of MPD. This finding replicates the results of various studies in different locations and with distinct epidemiological designs [7,12,15,31,33,34]. The prevalence ratios indicate that the frequency of MPD is over twice as high among workers exposed to unfavorable psychosocial working conditions. Siegrist [31], in a recent review of 16 studies, 12 longitudinal ones, covering a variety of occupations in various countries, showed that working in situations of high demand and low control or of effort/reward imbalance increase the risk of developing depression by up to eight times. Stansfeld [12], in a recent meta-analysis, also concluded that exposure to unfavorable psychosocial working environments, assessed by both scales, predict MPD.

Some studies using both scales concluded towards the higher strength of the ERI scale [15,30]. In this research, no relevant difference was found between the two models, being the magnitudes of the prevalence ratios slightly higher for the demand-control scale categories.

Social support may work as a moderator of the negative impact of stress in the worker's well-being and its absence can be associated with the existence of MPD [7,15,31,32]. In the studied population, the lack of social support at work was an important contributing factor for the presence of MPD, increasing their prevalence in approximately 60%, but changed only slightly the magnitude of the association observed with the subcategories of the demand-control model. These results are compatible with those found by other authors [15,16,32].

With regard to the interaction between social support at work and the demand-control model, the results of the few studies that tested such effect are quite inconsistent [14]. Such inconsistency was confirmed by Doef [29] in a 20-years review. The result in this study corroborates the absence of interaction between social support and psychosocial working demand.

Over-commitment is considered a moderating factor for the effect of working in situations with effort-reward imbalance, as well as an independent risk factor for MPD [15,26,30,31]. Among the workers studied, over-commitment was related with a two times increase in the prevalence of MPD. As far as we know, the interaction between effort-reward imbalance and over-commitment was investigated by few studies. Yu [15] found no interaction. Van Vegchel et al. [11] and Stansfeld et al. [12] pointed out in recent reviews that this third hypothesis of the ERI model still has no consistency. Our results suggest that overcommitted workers are less affected by the effort-reward imbalance at work. However, the consistency and meaning of such finding needs to be further investigated in other occupational groups and with different study designs.

Based on the results of this work and on the scientific evidences regarding the association between adverse psychosocial conditions at work and mental illness, a number of preventive measures can be recommended to reduce the burden of mental problems among bank workers [35]. They include increasing the number of workers where psychosocial job conditions are worse and prevalence of MPD is high, job rotation, reducing or eliminating the charging of production targets, improving pay conditions and flattening command.

## Conclusion

This study pointed out high prevalence of minor psychiatric disorders (MPD) among bank workers, reinforcing the evidence of the association between an unfavorable psychosocial working environment and these disorders. Prevalence of MPD was twice as high among workers exposed to high psychological demand and low control at work and under high effort and low reward working conditions. The lack of social support at work and the worker's over-commitment were also associated with a greater presence of MPD. Furthermore, a negative interaction between the Effort/Reward Imbalance and over-commitment was found.

The development of new studies among bank workers, especially of longitudinal design, may help to elucidate the mechanism of the associations found here. They should include workers from other banks - both private and public ones - so as to consider the whole diversity of the banking work in the country.

## List of abbreviations

ERI: Effort-rewarde Imbalance; JCQ: Job Content Questionnaire; MPD: Minor Psychiatric Disorders; GHQ: General Health Questionnaire; UFMG: Universidade Federal de Minas Gerais.

## Competing interests

The authors declare that they have no competing interests.

## Authors' contributions

LSS - design, data collection and analysis and paper writing.

SMB - design, data analysis and paper writing.

All authors read and approved the final manuscript

## Pre-publication history

The pre-publication history for this paper can be accessed here:

http://www.biomedcentral.com/1471-2458/10/686/prepub
